# The Fetus Can Teach Us: Oxygen and the Pulmonary Vasculature

**DOI:** 10.3390/children4080067

**Published:** 2017-08-03

**Authors:** Payam Vali, Satyan Lakshminrusimha

**Affiliations:** 1 University of California Davis School of Medicine, 2516 Stockton Blvd. Sacramento, CA 95817, USA; 2 Women and Children’s Hospital of Buffalo, 219 Bryant St., Buffalo, NY 14222, USA; slakshmi@buffalo.edu

**Keywords:** pulmonary hypertension of the newborn, oxygen target, oxygen saturation, fetal circulation

## Abstract

Neonates suffering from pulmonary hypertension of the newborn (PPHN) continue to represent an important proportion of patients requiring intensive neonatal care, and have an increased risk of morbidity and mortality. The human fetus has evolved to maintain a high pulmonary vascular resistance (PVR) in utero to allow the majority of the fetal circulation to bypass the lungs, which do not participate in gas exchange, towards the low resistance placenta. At birth, oxygen plays a major role in decreasing PVR to enhance pulmonary blood flow and establish the lungs as the organ of gas exchange. The failure of PVR to fall following birth results in PPHN, and oxygen remains the mainstay therapeutic intervention in the management of PPHN. Knowledge gaps on what constitutes the optimal oxygenation target leads to a wide variation in practices, and often leads to excessive oxygen use. Owing to the risk of oxygen toxicity, avoiding hyperoxemia is as important as avoiding hypoxemia in the management of PPHN. Current evidence supports maintaining arterial oxygen tension in the range of 50–80 mm Hg, and oxygen saturation between 90–97% in term infants with hypoxemic respiratory failure. Clinical studies evaluating the optimal oxygenation in the treatment of PPHN will be enthusiastically awaited.

## 1. Introduction

The recognition that the persistent elevation of pulmonary arterial pressures following birth leads to respiratory failure in the newborn was first described almost five decades ago [1]. Ongoing improvement in our understanding of the underlying pathogenesis leading to pulmonary hypertension in the past couple of decades has led to many novel therapeutic interventions targeting specific molecular cascades implicated in regulating the pulmonary vascular bed [2,3,4]. Pulmonary hypertension in the neonatal period can be broadly categorized into two groups: (1) failure of the elevated pulmonary vascular resistance to fall following birth, defined as persistent pulmonary hypertension of the newborn (PPHN), and (2) pulmonary hypertension as a consequence of severe pulmonary vascular disease in premature infants suffering from bronchopulmonary dysplasia [5]. PPHN is primarily seen in term and late preterm infants, reported in about 2 in every 1000 live-born infants [6], and has, also, been recognized in approximately 2% of premature infants with respiratory distress syndrome [7]. Despite improved neonatal care, early mortality from PPHN remains high with worse neurodevelopmental outcomes amongst the survivors [8,9]. Oxygen remains the mainstay therapeutic intervention in treating PPHN, although targeting the ideal oxyhemoglobin saturation (SO_2_) to avoid hypoxemia and prevent hyperoxemia remains to be determined. In the following article, we focus on reviewing the fetal vascular system, and defining the optimal oxygen target range in PPHN whereby the adverse effects of hyperoxia and hypoxia may be avoided in an attempt to decrease pulmonary vascular resistance and improve outcomes.

## 2. Fetal Pulmonary Vascular Resistance

During gestation, the fetus has evolved to divert a large proportion of the circulation away from the lungs towards the placenta, which serves as the organ of gas exchange, by maintaining an elevated pulmonary vascular resistance (PVR) and a low placental vascular resistance. The high PVR during the fetal period is due to a combination of mechanical factors, various vasoconstrictor mediators, and relative hypoxemia. The fetal small pulmonary arteries have a characteristic cuboidal endothelium and thick muscular coat [10,11], which contribute to the elevated PVR. Following birth, the rapid involution of the medial smooth muscle and the thinning of the small pulmonary arteries [12] play an important role in decreasing PVR. Other factors responsible in maintaining high PVR in utero include mechanical factors (compression of the small pulmonary arteries by fluid-filled alveoli and the lack of rhythmic distension) [13] and the interaction of vasoconstrictor (e.g., endothelin-1 and thromboxane) and vasodilator (e.g., prostacyclin and endothelium-derived nitric oxide) mediators on the pulmonary artery smooth muscles cells (PASMC) [14].

### 2.1. Effects of Oxygen and Fetal PVR

The distinguishing pulmonary vascular response to constrict in response to hypoxia (in contrast to the systemic arteries) was first recognized following experiments in cats in the 1940s [15]. Experiments in fetal lambs have shown that hypoxemia does not increase PVR at ≈70% gestation (100 out of 147–150 days full term gestation), whereas fetal hypoxemia at ≈90% gestation (132–138 days) doubles PVR [16]. A similar pattern is observed during fetal hyperoxemia, whereby a significant drop in PVR is observed in fetal lambs at 135 days gestation, while no change in PVR occurs in response to increased oxygen tension at 94–101 days gestation [17,18]. In human studies, providing 60% oxygen by face mask to expecting mothers between 20 and 26 weeks gestation did not alter fetal pulmonary blood flow, whereas an increase in pulmonary blood flow was appreciated at 31–36 weeks gestation [19].

The amount of blood pumped into the pulmonary circulation is dynamic and changes during fetal life. Early in gestation, the cross-sectional pulmonary vasculature is low, maintaining a high PVR, and the lungs receive only approximately 13% of the cardiac output at 20 weeks (canalicular stage of lung development), which increases to 25–30% at 30 weeks (saccular stage) owing to the proliferation of pulmonary vessels with a resultant fall in PVR, then drops to ≈16–21% near term gestation in response to active hypoxic pulmonary vasoconstriction secondary to the pulmonary vessels developing greater sensitivity to oxygen [20,21,22,23].

### 2.2. Fetal Circulation and the Role of the Lungs

**Figure 1:**
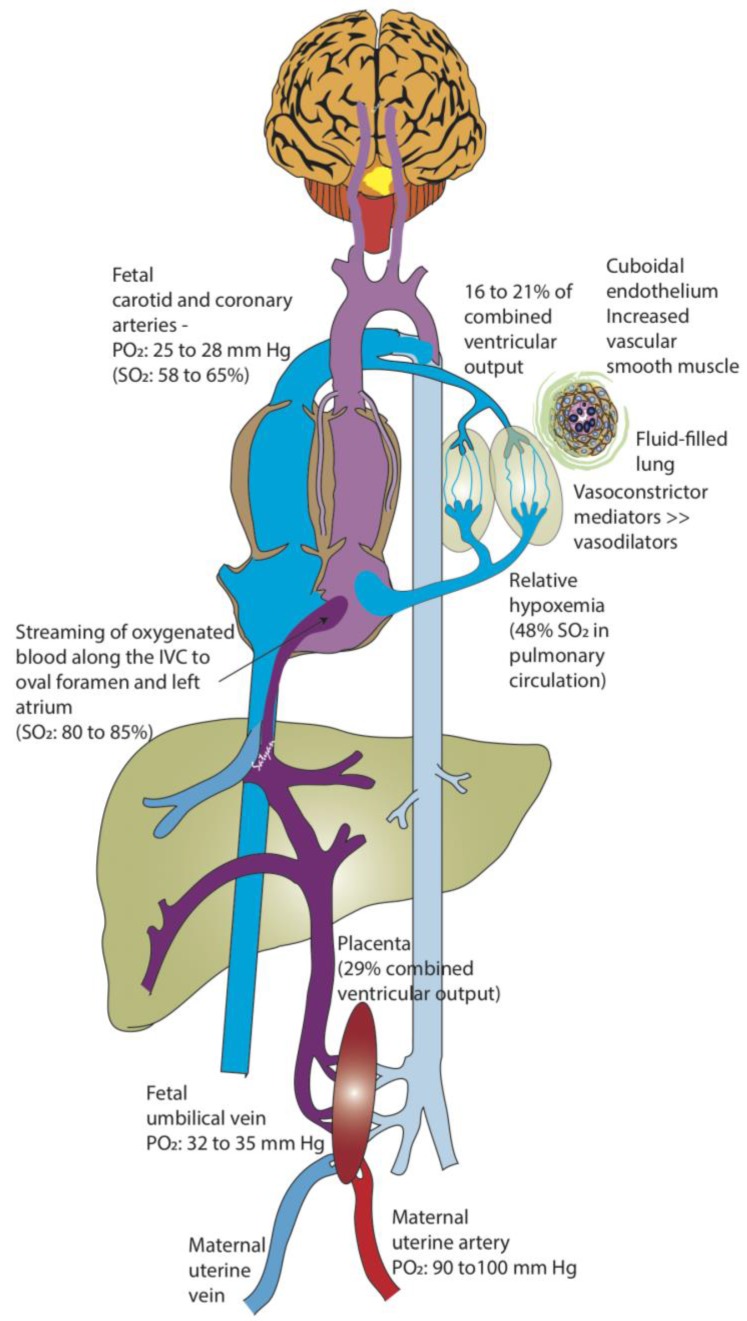
Fetal circulation. The placenta serves as a major buffer in reducing oxygen exposure to the fetus. The partial oxygen tension (PO_2_) in the maternal uterine artery is 90–100 mm Hg compared to 32–35 mm Hg in the fetal umbilical vein (UV). The relatively higher oxygenated UV blood does not completely mix with the blood returning from the fetal body in the inferior vena cava (IVC), and is preferentially streamed towards the left atrium (through the foramen ovale). As the lungs do not participate in gas exchange in utero, the fetal pulmonary vascular resistance is very high, and the pulmonary circulation only receives 16–21% of the combined ventricular cardiac output (by phase-contrast MRI and Doppler studies) in the near-term human fetus. As a result, there is only a small amount of desaturated blood from the pulmonary veins draining into the left atrium, maintaining a relatively high PO_2_ in the left heart. Therefore, the blood pumped into the aorta supplying the brain and coronaries contains the highest fetal PO_2_ (25–28 mm Hg: saturation 58% in human fetus and 65% in fetal lambs). Desaturated blood returning from the brain and the body into the right heart is pumped through the pulmonary artery and is mostly diverted through the ductus arteriosus to supply the rest of the body. Approximately 29–30% of the combined ventricular cardiac output circulates to the placenta. SO_2_: oxyhemoglobin saturation (Copyright Satyan Lakshminrusimha).

The purpose of the human fetal circulation is to enhance oxygenation to the fetal brain while minimizing toxicity. Owing to the fetus’s decreased oxygen consumption, in part due to its limited respiratory efforts and because thermoregulation is not necessary in utero, the fetus thrives in a hypoxic environment. The placenta serves as the primary buffer in limiting high blood oxygen exposure to the fetus by creating a large gradient between the maternal arterial partial pressure of oxygen PO_2_ = 90–100 mm Hg) and the umbilical vein (32–35 mm Hg) [20]. The placental (umbilical venous) and fetal pulmonary circulations comprise the sources of preload. Through an intricate system of shunts and streams, the higher blood oxygen content of the umbilical vein is preferentially diverted to the brain and coronary arteries (PO_2_ = 25–28 mm Hg, SO_2_ 58–65%; Figure 1) [20,24].

**Figure 2:**
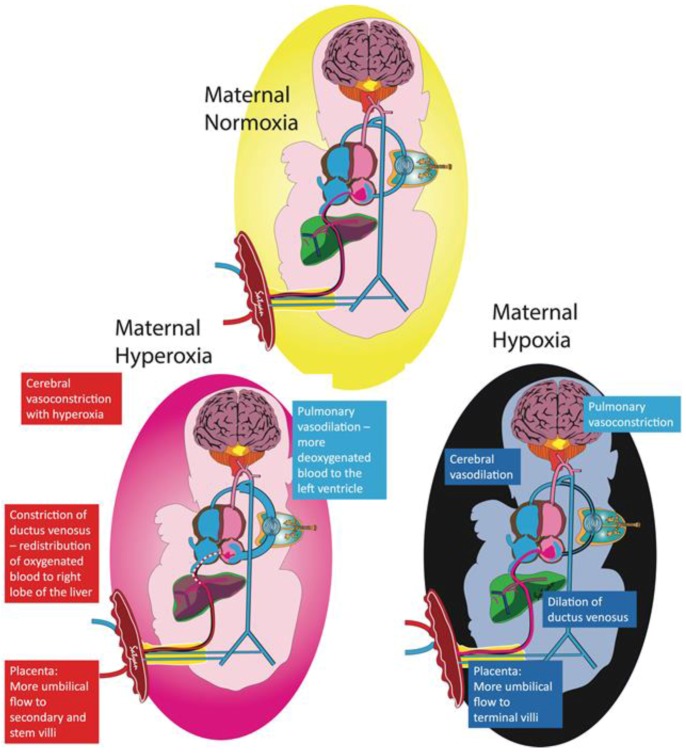
Fetal adaptation to maternal hypoxia and hyperoxia. The pulmonary circulation plays an important role in maintaining stable oxygen delivery to the brain. Exposing the mother to supraphysiologic levels of oxygen only slightly raises fetal umbilical venous (UV) partial oxygen tension (PO_2_). The higher fetal PO_2_ increases blood flow towards the lungs, resulting in more desaturated blood draining into the left atrium from the pulmonary veins, thus lowering the PO_2_ in the left heart supplying the brain. With more blood flowing to the lungs, there is decreased blood flow to the brain, effectively counterbalancing the higher UV PO_2_ and maintaining constant oxygen delivery to the brain. Other protective mechanisms to avoid oxygen toxicity are highlighted in the red boxes. Conversely, exposing the mother to a hypoxic environment leads to a decrease in UV PO_2_ causing increased pulmonary vascular resistance and less blood shunting to the lungs, therefore limiting the amount of desaturated blood returning to the left atrium from the pulmonary veins. Increased umbilical flow, dilation of the ductus venosus, and cerebral vasodilation increase blood flow to the brain to counteract the lower PO_2_ to maintain oxygen delivery (Copyright Satyan Lakshminrusimha).

The pulmonary circulation participates in maintaining the oxygen delivery to the brain within a narrow range by redirecting the pulmonary and systemic blood flows by altering the amount of blood shunting through the foramen ovale and ductus arteriosus. Lamb studies have shown that administering 100% oxygen to ewes raises uterine arterial PO_2_ to 400 mm Hg, while only increasing fetal umbilical venous PO_2_ to 40–50 mm Hg, and fetal ascending aorta PO_2_ to 30–35 mm Hg [20]. The marginal increase in PO_2_ in the fetal ascending aorta can be explained by (1) the diversion of blood from the terminal villi to secondary and stem villi in the placenta reducing oxygen uptake, (2) the constriction of the ductus venosus distributing blood to the right and left lobes of the liver [25], and (3) the higher fetal blood oxygen content decreasing PVR and increasing pulmonary blood flow [26], thus causing more desaturated blood to return from the pulmonary veins into the left atrium, effectively buffering the oxygen content in the left ventricle (Figure 2).

Conversely, during fetal hypoxemia, PVR increases and results in less blood flowing towards the pulmonary artery, which (1) reduces a further drop in PO_2_ in the left atrium by decreasing the amount of desaturated blood returning from the pulmonary veins, and (2) preferentially directs the umbilical vein blood through the foramen ovale into the left atrium and ultimately into the aorta, therefore successfully providing a higher oxygen delivery to the brain [27]. An inverse relationship between pulmonary flow and foramen ovale shunt has been demonstrated using phase-contrast MRI in late-gestation human fetuses [22].

### 2.3. Fetal Oxygenation

**Figure 3:**
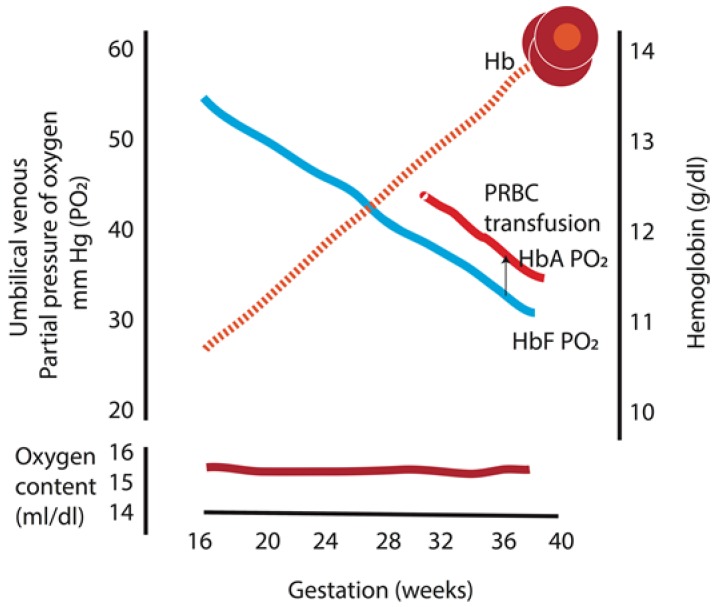
Umbilical venous partial pressure of oxygen and fetal hemoglobin during gestation. There is a linear decrease in the partial oxygen tension (PO_2_) with a concomitant rise in fetal hemoglobin as gestation progresses, which maintains the oxygen content in the blood constant throughout gestation. In addition, replacing fetal hemoglobin with adult hemoglobin packed red cells increases fetal PO_2_ by 4.8 mm Hg and maintains similar oxygen content. HbA: adult hemoglobin; HbF: fetal hemoglobin; PRBC: packed red blood cell. Data from [29] (Copyright Satyan Lakshminrusimha).

Experiments in sheep in the 1950s have demonstrated that there is a linear decrease in the umbilical vein PO_2_ without a change in maternal uterine artery PO_2_ as gestation progresses [28], which has, later, been confirmed in human studies (Figure 3) [29]. This seemingly counterintuitive drop in PO_2_ as the fetus is growing and increases its oxygen consumption can be better understood when considering the concomitant rise in hemoglobin that occurs during gestation. The fetus can maintain a constant oxygen content (and oxygen delivery) with a drop in PO_2_ while more hemoglobin is produced [29]. Replacing fetal hemoglobin by transfusing adult hemoglobin packed red blood cells increases fetal PO_2_ by ≈5 mm Hg and maintains a similar oxygen content (Figure 3) [29]. Therefore, the fetus avoids oxygen toxicity by keeping the oxygen tension in the blood low, and guarantees adequate oxygen delivery to meet tissue oxygen demand by maintaining a constant oxygen content.

**Figure 4:**
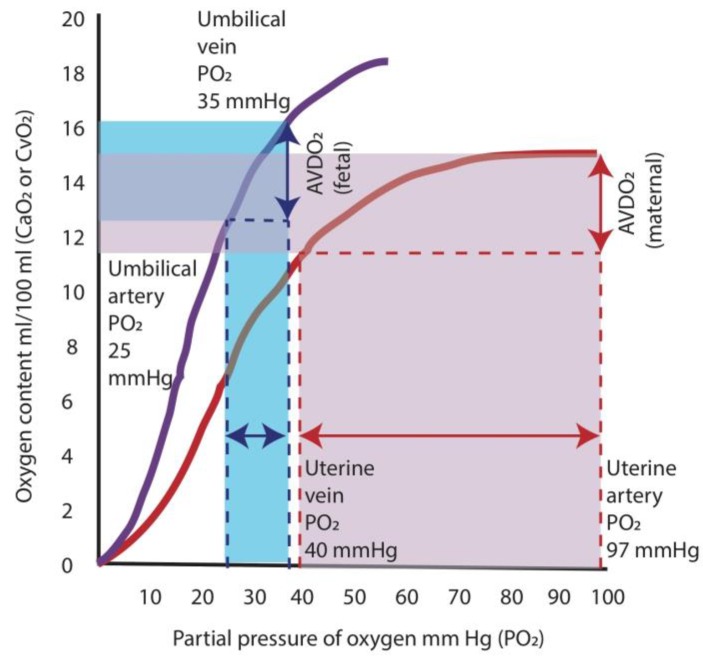
Oxygen hemoglobin dissociation curves and oxygen supply. The higher oxygen affinity of fetal hemoglobin (purple curve) shifts the oxygen dissociation curve to the left, which results in a greater release in oxygen at lower arterial partial oxygen tension (PO_2_) compared to adult hemoglobin (red curve). In the adult, a decrease in PO_2_ from 97 mm Hg (level present in arterial blood) to 40 mm Hg (level in venous blood) results in a release of oxygen amounting to ≈5 mL/dL (area shaded in red). For the fetus, the difference between the umbilical venous PO_2_ (35 mm Hg) and the umbilical arterial PO_2_ (25 mm Hg) results in a similar release of oxygen to the tissues of ≈4 mL/dL (area shaded in blue). AVDO_2_: arterio-venous difference in oxygen content. Data from [20] (Copyright Satyan Lakshminrusimha).

Properties of fetal hemoglobin also allow adequate oxygen supply to the tissues in the fetus. The higher oxygen affinity of fetal hemoglobin shifts the oxygen dissociation curve to the left, which results in a greater release in oxygen at lower arterial PO_2_ compared to adult hemoglobin. In the adult, a decrease in PO_2_ from 97 mm Hg (level present in arterial blood) to 40 mm Hg (level in venous blood) results in a release of oxygen amounting to ≈5 mL/dL. For the fetus, the difference between the umbilical venous PO_2_ (35 mm Hg) and the umbilical arterial PO_2_ (25 mm Hg) results in a similar release of oxygen to the tissues of ≈4 mL/dL (Figure 4) [20]. Therefore, the fetal oxygen extraction is similar to that of adults (at lower PO_2_).

## 3. Transition at Birth and PPHN

The most important trigger in reducing PVR at birth appears to be ventilation of the lungs and exposure to oxygen [30,31]. Oxygen is believed to be an important stimulus for the increased production of pulmonary endothelial nitric oxide (NO; a potent vasodilator) at the time of birth [32]. However, newborns with adverse in utero events or abnormalities of the pulmonary vascular bed that prevent a fall in PVR at birth will suffer from PPHN and hypoxic respiratory failure. Regardless of the underlying etiology responsible for PPHN, the accompanying hypoxemia, as a result of intrapulmonary shunting from ventilation/perfusion mismatch and/or extrapulmonary right-to-left shunting, further exacerbates the elevated PVR.

### The Role of Oxygen in Treating PPHN

As has been mentioned earlier, the pulmonary vessels of the fetus nearing term develop oxygen sensitivity and contract or relax in response to hypoxemia or hyperoxemia, respectively. PVR is predominantly regulated by the PASMC in precapillary resistance arterioles, but alveolar oxygen tension, however, exerts a greater effect on these vessels than PO_2_ [33,34]. The first study to describe the relationship between PO_2_ and PVR in a postnatal animal model conducted it on healthy newborn calves: a fall in PO_2_ below ≈45 mm Hg resulted in an abrupt increase in PVR [35]. Also, reducing arterial oxygen tension to fetal values in newborn lambs markedly increases PVR [36]. In a lamb PPHN ductal ligation and meconium aspiration asphyxia model, PVR steadily increases when PO_2_ falls below ≈60 mm Hg with a very steep increase in PVR at PO_2_ values below ≈14 mm Hg [37]. Furthermore, an increase from 50% to 100% inspired oxygen in this study did not produce any further decrease in pulmonary arterial pressure or PVR. Although lambs with PPHN that are resuscitated with 100% oxygen compared to 21% oxygen marginally enhance the decrease in PVR at birth, the effect is not sustained, and 100% oxygen induces oxidative stress and increases pulmonary artery reactivity [38,39]. Finally, PVR has been shown to increase when capillary SO_2_ falls below 85% or exceeds 98% [37], and PVR is lowest in the SO_2_ target range of 90–94%.

Since the identification of endothelium-derived relaxing factor as NO [40,41], inhaled nitric oxide (iNO) has become an indispensible drug (where available) in the treatment of pulmonary hypertension, including PPHN. Randomized clinical studies in term newborns with PPHN have shown a significant improvement in oxygenation as well as a reduction in the need for extracorporeal membrane oxygenation in the patients who were allocated to receive iNO [42,43,44,45]. However, as many as 40% of newborns with PPHN may not respond to iNO treatment, particularly newborns with congenital diaphragmatic hernia [45,46]. Superoxide anions, known to enhance pulmonary vasoconstriction, have been found to be twofold higher in lambs with PPHN [47], and have shown to inactivate NO to produce peroxynitrite [48,49]. Ventilation with 100% oxygen promotes the formation of reactive oxygen species (ROS), such as superoxide anions, that enhance vasoconstriction in the neonatal pulmonary circulation [48,50], and inactivate NO through the formation of peroxynitrite [48,51]. ROS have also been shown to cause pulmonary vasoconstriction by interfering with various enzymes of the NO pathway [52,53]. In addition, 100% oxygen use impairs subsequent vasodilation to iNO [54]. Owing to the severe adverse effects of superfluous oxygen use, possibly further exacerbated when combined with iNO due to the formation of peroxynitrite, avoiding hyperoxemia may be as important as avoiding hypoxemia in the management of PPHN.

## 4. Oxygen Use for PPHN in the Neonatal Intensive Care Unit (NICU)

The goal of oxygen therapy in PPHN is to (1) relax the pulmonary vasculature by decreasing PVR and to prevent hypoxemia, which would further exacerbate hypoxic pulmonary vasoconstriction, (2) provide adequate oxygen delivery to vital tissues such as the brain and heart while maintaining tissue oxygen demand, (3) avoid anaerobic metabolism and lactic acidosis, and (4) minimize oxidative stress. The evidence strongly suggests that hypoxemia and hyperoxemia can exacerbate hypoxic respiratory failure in PPHN, but the optimal SO_2_ range whereby a balance is achieved where either extreme can be avoided has not yet been established. In recent years, a great deal of interest has been gained in trying to determine the optimal SO_2_ target in premature infants, and despite large meta-analyses [55,56], no clear consensus has been reached regarding what constitutes the best and safest SO_2_ target range. Targeting oxyhemoglobin saturation in extreme premature infants is a balance of competing adverse outcomes (increased risk of necrotizing enterocolitis and death at the lower saturation ranges vs. increased risk of retinopathy of prematurity at the higher saturation range) [57]. Similar challenges in identifying the optimal SO_2_ in patients with PPHN are expected (worsening PVR and anaerobic metabolisms with low oxygen administration vs. oxidative stress with giving too much oxygen). However, there have been no clinical trials to date that have studied SO_2_ targets in term infants with lung disease.

A recent survey of neonatologist (492/1500 or 33% response rate) working in level 3 or 4 neonatal intensive care units (NICUs) across the USA evaluating oxygen management in neonates with PPHN has shown wide practice variations regarding the optimal oxyhemoglobin saturation or oxygen tension targets [58]. Seventy percent (70%) of respondents chose capillary SO_2_ targets > 95%, and 11% aimed to achieve arterial PO_2_ > 120 mm Hg, while as many as 6% preferred to treat with 100% oxygen until they were confident that the pulmonary vascular reactivity had stabilized and did not wean FIO_2_ despite SO_2_ of 100%. Only 28% reported using specific oxygen titration guidelines. In an international survey with a majority of the 200 respondents working in level 3 or 4 NICUs (96%) and having access to iNO (83%), 22% of respondents target arterial PO_2_ > 81 mm Hg, 38% target capillary SO_2_ > 96% (while 56% aim between 91 and 95%), and 80% target a hemoglobin level 13–15 g/dL [59]. The wide practice variation and the tendency to hyperoxygenate newborns with PPHN highlight the importance in studying the optimal oxygen target range for this patient population in the clinical setting.

### Determining the Optimal Oxygenation Range

The point at which hypoxic pulmonary vasoconstriction is evident should determine the lower limit of the target range for oxygenation. The PO_2_ surrounding the precapillary pulmonary arterioles (mainly influenced by alveolar PO_2_; PAO_2_) is the primary determinant of hypoxic pulmonary vasoconstriction [33,34]. However, as PAO_2_ is not directly measured in clinical settings, SO_2_ and arterial PO_2_ cut-offs have been evaluated in animal models, which have shown this point to correspond to an arterial PO_2_ of approximately 45–50 mmHg [4,35,37]. In lambs with PPHN induced by prenatal ligation of the ductus arteriosus, SO_2_ in the 90–97% range result in low PVR [4,37].

**Figure 5:**
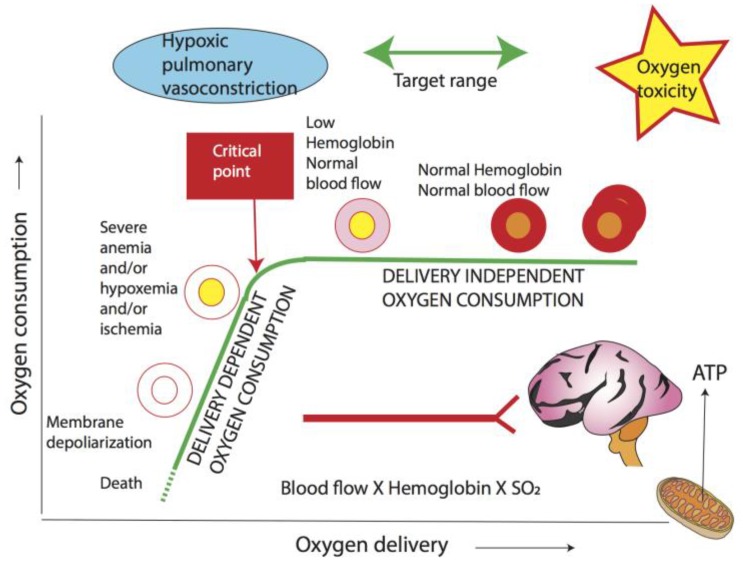
Relationship between oxygen delivery and consumption. O_2_ delivery is a product of blood flow and arterial O_2_ content. When O_2_ delivery decreases below a critical point, O_2_ consumption is compromised leading to anaerobic metabolism and lactic acidosis. The driving force for O_2_ into mitochondria is PO_2_. Increased mitochondrial PO_2_ can lead to reactive oxygen species formation, while hypoxia can exacerbate pulmonary vasoconstriction. The optimal target range encompasses oxygenation that guarantees an oxygen delivery higher than the critical point so that oxygen consumption is not dependent on delivery, while also avoiding oxygen toxicity. Hb: hemoglobin; SaO_2_: arterial oxyhemoglobin saturation (Copyright Satyan Lakshminrusimha).

The second factor determining the lower limit of target oxygenation is the critical point below which oxygen consumption decreases when oxygen delivery is reduced (Figure 5). SO_2_ is a crude measure of oxygenation, and is not the sole determinant in tissue oxygen delivery; an evaluation of the factors influencing oxygen content (hemoglobin level) and blood flow (cardiac output and pulmonary: systemic blood flow ratio) need to be considered to yield a better approach in treating PPHN. Similar to fetal life, arterial oxygen content in the newborn appears to be a key determinant of oxygen delivery, and extremely low levels of hemoglobin limit oxygen delivery to the tissues. Allowing placental transfusion at birth by delaying cord clamping to increase the newborn’s hemoglobin levels in infants at risk of PPHN, such as those with congenital diaphragmatic hernia, may improve oxygenation.

Defining an upper limit for target SO_2_ or arterial PO_2_ in the management of PPHN is more challenging, and would represent the level of oxygenation when toxicity develops. Studies in lambs and calves have shown that targeting arterial PO_2_ over 80–100 mm Hg does not result in additional pulmonary vasodilation. Furthermore, hyperoxia (arterial PO_2_ > 100 mm Hg) during the initial management of infants with hypoxic ischemic encephalopathy was associated with a poor neurodevelopmental outcome [60].

Finally, adequate blood flow to the tissues, especially the brain, is crucial to prevent neurodevelopmental impairment. Avoiding extremes of pH and arterial PCO_2_ remain necessary interventions in the overall management of PPHN. Acidemia has been shown to increase PVR [35], and hypocapnia is known to reduce cerebral blood flow and be associated with worse neurologic outcomes in perinatal asphyxia [61,62].

## 5. Summary

Oxygen therapy has long been considered a life sustaining intervention, and despite evidence from as early as the 1950s suggesting that oxygen can be toxic by generating free oxygen radicals [63], it has not been until recently that healthcare providers are taking a more conservative approach when administering oxygen. In the management of PPHN, avoiding hyperoxemia is as important as preventing hypoxemia. However, current knowledge gaps on what constitutes the optimal oxygenation target leads to a wide variation in practices amongst neonatologists, and often leads to excessive oxygen use. Current evidence, based mainly on data available from transitional models, supports maintaining arterial PO_2_ in the range of 50–80 mm Hg, and SO_2_ between 90 and 97% in term infants with hypoxemic respiratory failure. Clinical studies evaluating the optimal oxygenation in the treatment of PPHN will be enthusiastically awaited.

## References

[1] Gersony W., Duc G., Sinclair J. (1969). “Pfc syndrome” (persistence of the fetal circulation). Circulation.

[2] Aschner J.L., Gien J., Ambalavanan N., Kinsella J.P., Konduri G.G., Lakshminrusimha S., Saugstad O.D., Steinhorn R.H. (2016). Challenges, priorities and novel therapies for hypoxemic respiratory failure and pulmonary hypertension in the neonate. J. Perinatol..

[3] Steinhorn R.H. (2016). Advances in neonatal pulmonary hypertension. Neonatology.

[4] Lakshminrusimha S., Konduri G.G., Steinhorn R.H. (2016). Considerations in the management of hypoxemic respiratory failure and persistent pulmonary hypertension in term and late preterm neonates. J. Perinatol..

[5] Simonneau G., Gatzoulis M.A., Adatia I., Celermajer D., Denton C., Ghofrani A., Gomez Sanchez M.A., Krishna Kumar R., Landzberg M., Machado R.F. (2013). Updated clinical classification of pulmonary hypertension. J. Am. Coll. Cardiol..

[6] Walsh-Sukys M.C., Tyson J.E., Wright L.L., Bauer C.R., Korones S.B., Stevenson D.K., Verter J., Stoll B.J., Lemons J.A., Papile L.A. (2000). Persistent pulmonary hypertension of the newborn in the era before nitric oxide: Practice variation and outcomes. Pediatrics.

[7] Kumar V.H., Hutchison A.A., Lakshminrusimha S., Morin F.C., Wynn R.J., Ryan R.M. (2007). Characteristics of pulmonary hypertension in preterm neonates. J. Perinatol..

[8] Konduri G.G., Vohr B., Robertson C., Sokol G.M., Solimano A., Singer J., Ehrenkranz R.A., Singhal N., Wright L.L., Van Meurs K. (2007). Early inhaled nitric oxide therapy for term and near-term newborn infants with hypoxic respiratory failure: Neurodevelopmental follow-up. J. Pediatr..

[9] Porta N.F., Steinhorn R.H. (2012). Pulmonary vasodilator therapy in the NICU: Inhaled nitric oxide, sildenafil, and other pulmonary vasodilating agents. Clin. Perinatol..

[10] Hislop A., Reid L. (1972). Intra-pulmonary arterial development during fetal life-branching pattern and structure. J. Anat..

[11] Levin D.L., Rudolph A.M., Heymann M.A., Phibbs R.H. (1976). Morphological development of the pulmonary vascular bed in fetal lambs. Circulation.

[12] Hislop A., Reid L. (1973). Pulmonary arterial development during childhood: Branching pattern and structure. Thorax.

[13] Fineman J.R., Soifer S.J., Heymann M.A. (1995). Regulation of pulmonary vascular tone in the perinatal period. Annu. Rev. Physiol..

[14] Lakshminrusimha S., Steinhorn R.H. (1999). Pulmonary vascular biology during neonatal transition. Clin. Perinatol..

[15] Von Euler U., Liljestrand G. (1946). Observations on the pulmonary arterial blood pressure in the cat. Acta Physiol. Scand..

[16] Lewis A.B., Heymann M.A., Rudolph A.M. (1976). Gestational changes in pulmonary vascular responses in fetal lambs in utero. Circ. Res..

[17] Morin F.C., Egan E.A. (1992). Pulmonary hemodynamics in fetal lambs during development at normal and increased oxygen tension. J. Appl. Physiol. (1985).

[18] Morin F.C., Egan E.A., Ferguson W., Lundgren C.E. (1988). Development of pulmonary vascular response to oxygen. Am. J. Physiol..

[19] Rasanen J., Wood D.C., Debbs R.H., Cohen J., Weiner S., Huhta J.C. (1998). Reactivity of the human fetal pulmonary circulation to maternal hyperoxygenation increases during the second half of pregnancy: A randomized study. Circulation.

[20] Rudolph A. (2009). The fetal circulation. Congenital Diseases of the Heart: Clinical-Physiological Considerations.

[21] Rasanen J., Wood D.C., Weiner S., Ludomirski A., Huhta J.C. (1996). Role of the pulmonary circulation in the distribution of human fetal cardiac output during the second half of pregnancy. Circulation.

[22] Prsa M., Sun L., van Amerom J., Yoo S.J., Grosse-Wortmann L., Jaeggi E., Macgowan C., Seed M. (2014). Reference ranges of blood flow in the major vessels of the normal human fetal circulation at term by phase-contrast magnetic resonance imaging. Circ. Cardiovasc. Imaging.

[23] Kinsella J.P., Ivy D.D., Abman S.H. (1994). Ontogeny of no activity and response to inhaled no in the developing ovine pulmonary circulation. Am. J. Physiol..

[24] Lakshminrusimha S. (2012). The pulmonary circulation in neonatal respiratory failure. Clin. Perinatol..

[25] Sorensen A., Holm D., Pedersen M., Tietze A., Stausbol-Gron B., Duus L., Uldbjerg N. (2011). Left-right difference in fetal liver oxygenation during hypoxia estimated by bold mri in a fetal sheep model. Ultras. Obstet. Gynecol..

[26] Konduri G.G., Gervasio C.T., Theodorou A.A. (1993). Role of adenosine triphosphate and adenosine in oxygen-induced pulmonary vasodilation in fetal lambs. Pediatr. Res..

[27] Sun L., Macgowan C.K., Sled J.G., Yoo S.J., Manlhiot C., Porayette P., Grosse-Wortmann L., Jaeggi E., McCrindle B.W., Kingdom J. (2015). Reduced fetal cerebral oxygen consumption is associated with smaller brain size in fetuses with congenital heart disease. Circulation.

[28] Barron D.H., Alexander G. (1952). Supplementary observations on the oxygen pressure gradient between the maternal and fetal bloods of sheep. Yale J. Biol. Med..

[29] Soothill P.W., Nicolaides K.H., Rodeck C.H., Campbell S. (1986). Effect of gestational age on fetal and intervillous blood gas and acid-base values in human pregnancy. Fetal. Ther..

[30] Teitel D.F., Iwamoto H.S., Rudolph A.M. (1990). Changes in the pulmonary circulation during birth-related events. Pediatr. Res..

[31] Reid D.L., Thornburg K.L. (1990). Pulmonary pressure-flow relationships in the fetal lamb during in utero ventilation. J. Appl. Physiol. (1985).

[32] Shaul P.W., Farrar M.A., Zellers T.M. (1992). Oxygen modulates endothelium-derived relaxing factor production in fetal pulmonary arteries. Am. J. Physiol..

[33] Kato M., Staub N.C. (1966). Response of small pulmonary arteries to unilobar hypoxia and hypercapnia. Circ. Res..

[34] Moudgil R., Michelakis E.D., Archer S.L. (2005). Hypoxic pulmonary vasoconstriction. J. Appl. Physiol. (1985).

[35] Rudolph A.M., Yuan S. (1966). Response of the pulmonary vasculature to hypoxia and h+ ion concentration changes. J. Clin. Investig..

[36] Rudolph A.M. (1979). Fetal and neonatal pulmonary circulation. Annu. Rev. Physiol..

[37] Lakshminrusimha S., Swartz D.D., Gugino S.F., Ma C.X., Wynn K.A., Ryan R.M., Russell J.A., Steinhorn R.H. (2009). Oxygen concentration and pulmonary hemodynamics in newborn lambs with pulmonary hypertension. Pediatr. Res..

[38] Lakshminrusimha S., Steinhorn R.H., Wedgwood S., Savorgnan F., Nair J., Mathew B., Gugino S.F., Russell J.A., Swartz D.D. (2011). Pulmonary hemodynamics and vascular reactivity in asphyxiated term lambs resuscitated with 21 and 100% oxygen. J. Appl. Physiol. (1985).

[39] Lakshminrusimha S., Russell J.A., Steinhorn R.H., Ryan R.M., Gugino S.F., Morin F.C., Swartz D.D., Kumar V.H. (2006). Pulmonary arterial contractility in neonatal lambs increases with 100% oxygen resuscitation. Pediatr. Res..

[40] Ignarro L.J., Byrns R.E., Buga G.M., Wood K.S. (1987). Endothelium-derived relaxing factor from pulmonary artery and vein possesses pharmacologic and chemical properties identical to those of nitric oxide radical. Circ. Res..

[41] Palmer R.M., Ferrige A.G., Moncada S. (1987). Nitric oxide release accounts for the biological activity of endothelium-derived relaxing factor. Nature.

[42] Neonatal Inhaled Nitric Oxide Study Group (1997). Inhaled nitric oxide in full-term and nearly full-term infants with hypoxic respiratory failure. N. Engl. J. Med..

[43] Davidson D., Barefield E.S., Kattwinkel J., Dudell G., Damask M., Straube R., Rhines J., Chang C.T. (1998). Inhaled nitric oxide for the early treatment of persistent pulmonary hypertension of the term newborn: A randomized, double-masked, placebo-controlled, dose-response, multicenter study. Pediatrics.

[44] Clark R.H., Kueser T.J., Walker M.W., Southgate W.M., Huckaby J.L., Perez J.A., Roy B.J., Keszler M., Kinsella J.P. (2000). Low-dose nitric oxide therapy for persistent pulmonary hypertension of the newborn. N. Engl. J. Med..

[45] Roberts J.D., Fineman J.R., Morin F.C., Shaul P.W., Rimar S., Schreiber M.D., Polin R.A., Zwass M.S., Zayek M.M., Gross I. (1997). Inhaled nitric oxide and persistent pulmonary hypertension of the newborn. N. Engl. J. Med..

[46] The Neonatal Inhaled Nitric Oxide Study Group (NINOS) (1997). Inhaled nitric oxide and hypoxic respiratory failure in infants with congenital diaphragmatic hernia. Pediatrics.

[47] Brennan L.A., Steinhorn R.H., Wedgwood S., Mata-Greenwood E., Roark E.A., Russell J.A., Black S.M. (2003). Increased superoxide generation is associated with pulmonary hypertension in fetal lambs: A role for nadph oxidase. Circ. Res..

[48] Lakshminrusimha S., Russell J.A., Wedgwood S., Gugino S.F., Kazzaz J.A., Davis J.M., Steinhorn R.H. (2006). Superoxide dismutase improves oxygenation and reduces oxidation in neonatal pulmonary hypertension. Am. J. Respir. Crit. Care. Med..

[49] Faraci F.M., Didion S.P. (2004). Vascular protection: Superoxide dismutase isoforms in the vessel wall. Arterioscler. Thromb. Vasc. Biol..

[50] Sanderud J., Norstein J., Saugstad O.D. (1991). Reactive oxygen metabolites produce pulmonary vasoconstriction in young pigs. Pediatr. Res..

[51] Belik J., Jankov R.P., Pan J., Yi M., Chaudhry I., Tanswell A.K. (2004). Chronic o2 exposure in the newborn rat results in decreased pulmonary arterial nitric oxide release and altered smooth muscle response to isoprostane. J. Appl. Physiol. (1985).

[52] Farrow K.N., Groh B.S., Schumacker P.T., Lakshminrusimha S., Czech L., Gugino S.F., Russell J.A., Steinhorn R.H. (2008). Hyperoxia increases phosphodiesterase 5 expression and activity in ovine fetal pulmonary artery smooth muscle cells. Circ. Res..

[53] Sanderud J., Oroszlàn G., Bjøro K., Kumlin M., Saugstad O.D. (1995). D-penicillamine inhibits the action of reactive oxygen species in the pig pulmonary circulation. J. Perinat. Med..

[54] Lakshminrusimha S., Russell J.A., Steinhorn R.H., Swartz D.D., Ryan R.M., Gugino S.F., Wynn K.A., Kumar V.H., Mathew B., Kirmani K. (2007). Pulmonary hemodynamics in neonatal lambs resuscitated with 21%, 50%, and 100% oxygen. Pediatr. Res..

[55] Saugstad O.D., Aune D. (2014). Optimal oxygenation of extremely low birth weight infants: A meta-analysis and systematic review of the oxygen saturation target studies. Neonatology.

[56] Manja V., Lakshminrusimha S., Cook D.J. (2015). Oxygen saturation target range for extremely preterm infants: A systematic review and meta-analysis. JAMA Pediatr..

[57] Cummings J.J., Lakshminrusimha S., Polin R.A. (2016). Oxygen-saturation targets in preterm infants. N. Eng. J. Med..

[58] Alapati D., Jassar R., Shaffer T.H. (2017). Management of supplemental oxygen for infants with persistent pulmonary hypertension of newborn: A survey. Am. J. Perinatol..

[59] Nakwan N., Chaiwiriyawong P. (2016). An international survey on persistent pulmonary hypertension of the newborn: A need for an evidence-based management. J. Neonatal. Perinatal. Med..

[60] Kapadia V.S., Chalak L.F., DuPont T.L., Rollins N.K., Brion L.P., Wyckoff M.H. (2013). Perinatal asphyxia with hyperoxemia within the first hour of life is associated with moderate to severe hypoxic-ischemic encephalopathy. J. Pediatr..

[61] Stiris T., Odden J.P., Hansen T.W., Hall C., Bratlid D. (1989). The effect of arterial pco2-variations on ocular and cerebral blood flow in the newborn piglet. Pediatr. Res..

[62] Pappas A., Shankaran S., Laptook A.R., Langer J.C., Bara R., Ehrenkranz R.A., Goldberg R.N., Das A., Higgins R.D., Tyson J.E. (2011). Hypocarbia and adverse outcome in neonatal hypoxic-ischemic encephalopathy. J. Pediatr..

[63] Gerschman R., Gilbert D.L., Nye S.W., Dwyer P., Fenn W.O. (1954). Oxygen poisoning and x-irradiation: A mechanism in common. Science.

